# Supplementary material for: Are carbon emissions trading and green financial instruments synergistic? -Comprehensive quantitative research based on content analysis
Source: PLoS One. 2024 Mar 7;19(3):e0298601. doi: 10.1371/journal.pone.0298601 (PMC10919601; doi:10.1371/journal.pone.0298601)
Supplement: S1 Data — (ZIP) [file pone.0298601.s001.zip › Data/地级市Z_F工作报告.zip!/║■▒▒/╢⌡╓▌╩╨/└┤╫╘╣1⁄2╓┌║┼í╛┬φ┐╦╩2╛▌═°í┐_2013.docx]

各位代表：现在，我代表市人民政府向大会作政府工作报告，请予审议，并请各位市政协委员和列席人员提出意见。

　　一、2012年工作回顾刚刚过去的2012年，是极不平凡的一年，受国内外大气候影响，我市国民经济遇到了前所未有的困难。面对经济下行压力，全市上下以科学发展观为指导，按照省委、省政府和市委的决策部署，团结拼搏、改革创新、难中求进，实现了经济平稳较快增长，保持了社会和谐稳定，“五个鄂州”建设实现了良好开局。

　　———经济实力再上台阶。预计全年，实现地区生产总值560亿元，增长12％，人均生产总值首次超过8000美元；全社会固定资产投资450亿元，增长33.6%；规模以上工业增加值实现300亿元，增长16.6%；财政总收入突破50亿元大关，达到50.17亿元，增长21.3%；地方公共预算收入32.58亿元，增长23.8%；全社会消费品零售总额181亿元，增长15.8%；招商引资总额270亿元，增长50%，实际利用外资1.65亿美元，增长19.2%；城镇居民人均可支配收入19466元，增长14.5%；农民人均纯收入9072元，增长14.7%。主要指标增幅均高于全省平均水平。

　　———产业结构不断优化。工业经济实力增强。大力实施“五大工程”，科学安排调度各项资金帮助企业渡难关。规模以上工业实现销售收入首次跨上千亿台阶，达到1040亿元。

　　新增收入过亿元企业45家，净增规模以上企业37家。顾地科技公司成功上市。高新技术产业发展加快。新增高新技术企业18家，新增高新技术产品企业31家，科技加速器、物联网等重大高新项目成功落户。高新技术产业增加值占GDP的比重达到13.6%，提高1.3个百分点。高新技术产业对经济增长的贡献率提高1.18个百分点。与此同时，冶金、电力等传统产业占比下降。第三产业已经成为我市经济新的增长点。引进建设了武汉东家居城、豪威广场、航宇商贸城、海宁皮草城等大型商贸项目。休闲旅游产业发展加快,成功举办了第十三届梁子湖捕鱼旅游节,全市实现旅游业总收入31.9亿元，增长29%。金融机构存款余额345.23亿元，增长16.3%；贷款余额225亿元，增长30.5%，贷存比达到65.17%，居全省前列，服务实体经济的作用加强，连续六年被省政府授予“信用鄂州”称号。节能减排效果明显。实施节能减排、循环经济项目76个，关停高能耗、高污染“五小”企业400余家，淘汰落后钢铁产能147万吨。单位生产总值综合能耗下降5%，二氧化碳和主要污染物排放均低于省控目标，总体环境质量有所改善。

　　———发展后劲显著增强。基础设施建设投入大幅增加。完成各类基础设施建设投入78.7亿元，开工项目、工程量、投资额均创历史新高。主城区路网、市政基础设施建设投入13亿元，电网建设改造投资8亿元，均创历年之最。鄂东交通枢纽建设顺利推进。公铁水交通设施完成投资54.9亿元。汉鄂高速、大广南鄂州段、鄂东大道、凤莲大道、东沟大桥建成通车，武汉至葛店开发区301路公交车投入运营。武汉新港三江港区综合码头、吴楚大道、鄂黄高速以及武黄、武冈城际铁路建设进展顺利，鄂州火车站主站房春节前可投入使用。

　　园区建设规模超历史。全市园区基础设施建设投资超过18亿元，是投入最多的一年。葛店开发区升级为国家级开发区；鄂州开发区同时启动20个基础设施项目建设；花湖开发区、红莲湖旅游度假区、梧桐湖新城、三江港区基础设施建设力度加大。全市基本形成以国家级开发区为龙头，以省级开发区为支撑，以各区园区为节点，错位发展、优势互补的区域经济发展格局。招商引资和项目建设成效显著。

　　新签约亿元项目117个，在建亿元项目137个。

　　纳入市级调度的重点项目完成投资302亿元。

　　顾地总部、长动铸锻等重点在建项目进展顺利，金牛管业、华工科技等39个项目竣工。洋澜湖综合治理、鄂州职业大学建设利用德国政府贷款4900万欧元取得实质进展。

　　———综合改革不断深入。行政层级和审批制度改革实现新突破。启动葛店开发区减少行政层级改革试点，撤销葛店镇并入葛店开发区，汀祖经济发达镇改革试点加快推进。

　　建立了市场准入和项目建设“绿色通道”，推进行政审批“三集中三到位”，实现了全省市州审批项目最少、时限最短、收费最低的“三最”目标。农村产权制度改革取得新进展。出台《关于加快推进农村产权制度改革的指导意见》，成立市农村综合产权交易所，实现建设用地指标交易。组建市农业发展投资公司，搭建农村产权融资平台。社会管理创新开创新局面。探索农村示范社区建设，推进电子政务进社区，33项行政服务实现社区网上办理，启用全市统一的行政确认专用章。将全市城乡划分为3462个管理网格，实行社会化、信息化、精细化管理，基本形成覆盖全市的新型社会管理模式。医疗卫生改革惠及群众。市直4家公立医院在全省率先取消药品加成，组建市中心医院医疗集团，城乡公共卫生服务体系信息化、标准化建设取得明显成效。其他各项改革试点取得新成果。事业单位改革、廉政风险预警防控试点、房地产税制改革试点、居民家庭经济状况核对试点、节水型社会建设试点、“三网”融合试点等11项国家和省级改革试点工作都取得阶段性成果。

　　———城乡一体化深入推进。城乡生产生活条件进一步改善。进一步落实巩固“厅市共建”合作成果，争取项目资金7.6亿元。农村公路、安全饮水、环境连片整治、电网改造、国土整治、扶贫开发、沼气工程等设施建设顺利推进。投资1.18亿元完成花马湖、洋澜湖等泵站更新改造。“三万”活动成果进一步巩固和扩大，完成塘堰改造2498口，深入推进“清洁乡村”工程，城乡一体的生活垃圾收集处理体系正在建立。特色镇和新社区建设进一步加快。

　　在加快建设63个农村新社区的基础上，筹资2亿元支持特色镇建设，集镇功能更加完善。城乡基本公共服务进一步均衡。在“完善、扩面、提高”上下功夫，强力推动城市交通、供水、供电等基础设施向农村深度延伸，公共服务能力进一步提高。现代都市农业进一步壮大。围绕水产、蔬菜、畜禽、林果四大支柱产业，加快发展观光农业、休闲度假等都市农业新业态。

　　新增农业产业化经营组织26家、农民专业合作组织106家、省级龙头企业7家。一批加工企业入驻华容省级农产品加工园。全市实现农业增加值66.5亿元，同比增长5%。水产业产值突破百亿元，畜禽规模化养殖率居全省前列。

　　粮食产量实现“九连增”。

　　———社会事业全面进步。围绕幸福鄂州建设，不断加大民生领域投入，民生支出占地方公共财政支出比重达到67%。投资12.16亿元的“十件实事”全面完成。推进以创业带动就业。新增个体工商户6184户，创办小微企业740家，新增城镇就业2.35万人，组织农业劳动力转移就业1.27万人。社会保障和救助体系不断完善。城乡居民养老保险和医保参保率分别保持在90%和99%以上；社保“一卡通”工程启动运行；企业退休人员养老金人均提高211元；对城乡低保继续提标扩面；新改扩建葛店、碧石等9所农村福利院；为全市1178名90岁以上老人发放高龄补贴；落实残疾人特惠政策，惠及1.2万名贫困残疾人；特殊困难人群救助工作加强。住房保障能力提升。

　　建成各类保障性住房7650套；对1330户困难户发放住房租赁补贴。科教兴市成效显著。争取上级教育专项资金3000万元，积极开展义务教育均衡发展改革试验，鄂城区率先通过省评估验收；市实验小学建成招生；鄂州高中高考9名学生考入清华、北大，创历史最佳成绩，并获得北大“中学校长实名推荐”资格；启动鄂州职业大学国家骨干职教院校建设。落实省级以上科技专项34个,专利申请量达到1220件;技术交易额首次超亿元。文化惠民工程强力推进。

　　京剧二团等6家单位完成转企改制；大型文艺演出增加，群众性文化体育活动蓬勃发展；大型舞蹈诗《吴都风华》获得湖北省首届艺术节五个单项奖；总投资1.5亿元的鄂州市博物馆建成揭牌。市中医院改扩建工程竣工，疾病预防控制取得明显成效，及时控制手足口病和波及我市的二号病。“阳光计生”、“幸福人家促进计划”等人口计生工作取得成效。物价继续保持平稳，居民消费价格指数上涨2.9%。

　　加强法律社会服务，积极开展矛盾纠纷排查化解，着力解决信访突出问题。以硬措施改善软环境，深入推进治庸问责。加大生产质量监督、流通环节监管和食品药品监管力度，治安和刑事案件下降，社会治安形势持续好转，安全生产形势稳定，人民满意度上升。

　　与此同时，统计、粮食、供销、民族宗教、外事侨务、对台、保密、档案方志、工会、青年、妇女儿童、机关事务、国资管理、保险、邮政、通讯、石油石化、烟草、无线电、广播电影电视、新闻出版、社会科学、人防、消防、气象、国防教育动员和对口援建等工作都取得了新的成绩。

　　各位代表，在宏观经济形势严峻，困难压力增多的情况下取得这样的成绩，确实来之不易。这来自于市委的坚强领导，来自于各方的鼎力支持，来自于全市人民的团结拼搏。借此机会，我谨代表市人民政府向辛勤工作在各个岗位的广大干部群众，向各位人大代表、政协委员，向各民主党派、工商联、无党派、人民团体和社会各界人士，向为鄂州发展做出贡献的老同志，向驻军指战员、武警官兵、公安干警以及所有关心支持参与鄂州改革发展的海内外朋友表示崇高的敬意和衷心的感谢！在充分肯定成绩的同时，我们也清醒地看到,发展不够仍然是鄂州最大的实际。经济总量偏小、综合实力不强、产业层次偏低、传统产业比重过大的现状没有得到根本改变，一些深层次矛盾还有待进一步化解。当年地区生产总值、规模以上工业增加值等指标增幅未完成市七届人大一次会议确定的目标。逆水行舟不进则退，百舸争流慢进亦退。我们必须进一步增强忧患意识，始终保持清醒头脑，更加主动地应对困难，更加周密地谋划措施，更加积极地抓好落实，以高度的责任感和使命感，把今年的各项工作抓好。

　　二、2013年工作安排今年是深入贯彻落实党的十八大精神的开局之年，是实施“十二五”规划承前启后的关键一年，我们将迎来建市30周年，做好今年的政府工作，具有十分重要的意义。

　　今年政府工作的总体要求是：深入贯彻党的十八大精神，以邓小平理论、“三个代表”重要思想、科学发展观为指导，围绕建设“五个鄂州”的主要任务和建设宜居宜业组群式大城市的奋斗目标，以提高经济增长质量和效益为中心，按照“竞进提质”的总要求，深入推进综合改革，加快城乡一体化发展，着力稳增长、调结构、转方式、强体系、惠民生，努力保持经济持续健康较快发展和社会和谐稳定，以优异成绩迎接建市30周年。

　　今年全市经济社会发展的主要预期目标是：地区生产总值增长12%；全社会固定资产投资增长25%；规模以上工业增加值增长16%；地方公共财政预算收入增长15%；社会消费品零售总额增长15%；外贸出口增长8%；招商引资总额增长35%，其中实际利用外资增长10%；城镇居民人均可支配收入增长12%；农村居民人均纯收入增长13%；城镇新增就业2万人，城镇登记失业率控制在4.5%以内；居民消费价格指数与省保持一致；人口自然增长率、单位生产总值综合能耗、二氧化碳、主要污染物排放总量均控制在省下达目标之内。

　　围绕以上目标，重点抓好七个方面的工作。

　　（一）坚持稳增长扩规模，壮大经济总量。发展仍是解决所有问题的关键，在相当长的一个时期，做大规模仍然是鄂州经济发展的头等大事。一是加快推进项目建设。坚持扩大投资与优化投向并重，继续实施“三个一百工程”，抓好重大项目建设，力争续建和新开工项目、招商引资亿元项目、中省投资项目分别超百个，确保全年投资完成560亿元。加快重大工业项目建设。继续抓好“五大工程”，更好的发挥财政性资金的引导作用，加强生产要素的保障协调，重点推进鄂钢大棒、波尔亚太三期、顾地总部、金牛管业二期、兴欣建材三期、长动铸锻、华扬太阳能等重点工业项目建设，力争新增规模以上工业企业30家。加强项目谋划储备。立足现有基础，围绕国家产业导向，积极策划和申报一批关联度高、带动力强的重大支撑性项目。加快重大项目前期工作力度，推进鄂州电厂三期进程，确保三江港综合码头、富地LNG上半年开工，启动中海油清洁能源产业基地前期建设。加强重点项目协调服务，优化项目建设外部环境。培育和壮大市场主体。进一步贯彻落实省、市推动民营经济发展的政策措施，推动民营企业跨越发展。二是全力抓好招商引资。在继续扶持现有企业做大做强的同时，围绕高新技术产业和新兴产业，突出产业招商、园区招商，提升招商引资的质量和水平。紧盯有意向的大集团、大企业、大项目、好项目，落实项目论证制度，提高项目质量。将投资强度、高新产业占比纳入招商工作考核体系。坚持集约节约用地，项目投资强度平均每亩要达到160万元以上。葛店开发区作为国家级开发区，引进高新技术和战略性新兴产业的比例要力争达到50%以上。三是加快园区平台建设。按产城一体的格局加快推进园区建设，完善功能布局，促进要素汇聚。继续推进鄂州开发区18平方公里集中连片以及各区10平方公里园区配套建设。放大葛店国家级经济开发区的综合效应，探索“园外园”运作模式，落实“飞地经济”的相关政策，推动园区共建共享，引导产业集中集成。四是突破性发展第三产业。加快发展现代物流等生产性服务业，探索以“地主港”开发模式，加快三江港区整体规划与开发，按照千亿元产业目标，引进战略投资者开发仓储、配送、多式联运等生产性物流体系，努力打造长江中游区域性物流中转基地。精心培育上市后备企业，全力支持大通公司尽快“过会”上市。培育金融服务市场，鼓励民间金融市场规范发展，引进股份制商业银行来鄂州开设分支机构。全面实施中小商贸企业成长工程，确保新增限上商贸企业30家。提升旅游休闲产业。搭建投资平台，加快大梁子湖生态旅游度假区建设和西山风景区保护开发，打造三国吴都文化旅游和湖泊生态水乡旅游精品。大力发展文化产业。推进梧桐湖创意产业园建设，打造一批展示深厚吴楚文化底蕴的项目。

　　（二）加快调结构转方式，提升发展质量。在保持经济较快发展的同时，积极调整经济结构，转变发展方式。一是加速传统产业改造升级。推进信息化与工业化融合。支持企业运用信息技术嫁接改造传统的生产工艺、技术装备和经营管理，逐步实现生产过程数控化、企业管理信息化。积极争取中省技改专项资金，支持鄂钢、电厂、世纪新峰等骨干企业提质改造。以先进适用技术激活现有存量，推进传统产业高端化、传统产品新型化。加快推进传统产业链整合。发挥政府与市场的引导作用和企业的产权纽带作用，重点推动建材、冶金等行业产能整合、上下游融合、市场营销联合，营造我市传统产业新优势。大力实施质量兴市战略，加快培育一批知名品牌。二是加快发展新兴产业。研究推进战略性新兴产业发展的政策措施，设立1000万元高新产业引导资金，扶持高新产业形成气候。推进葛店开发区与东湖高新区的产业对接，形成区域性、一体化、产学研融合型高新产业板块。加快物联网产业园、华中数控、华工科技三期、镁锂镁合金等高新项目建设，力争葛店科技企业加速器一期项目竣工，确保全市新增高新技术企业20家、高新产业增加值占GDP比重高于全省平均水平。三是提高自主创新能力。发挥企业主体作用，推动产学研合作，建立高层次、专业性企业技术中心、工程技术中心，引导企业提高研发经费投入。完善科技创新投融资体系和扶持机制，引进培育风险投资平台。加快太阳能、节能建材、金刚石超硬材料三个国家级质量中心等创新载体建设，争取更多的院士工作站、博士后产业基地落户我市。推动新技术、新工艺和新产品的开发应用，加速实现产业化。积极创建国家知识产权示范城市，培育一批具有自主知识产权、有核心竞争力的创新型龙头企业。四是加快发展循环经济。加快“青阳鄂”循环经济示范区建设，重点打造清洁能源、冶金绿色高效生产、机电装备再制造、新型节能环保建材、废弃物综合利用等循环经济产业链，加快鄂钢、电厂等企业余能、废弃物综合利用和中再生项目建设。实施减排工程，严格执行节能评价和环境影响评价制度，严控高污染、低能效项目，继续淘汰落后产能。

　　（三）突出新型城镇化引领，推进城乡统筹发展。我市城乡一体化已进入新的发展阶段，要按照“五年大变化”的要求，以新型城镇化引领全市城乡一体化发展。一是加快推进农村城镇化。按照以主城为龙头、新城为支撑、特色镇为链接、农村新社区为基础的“四位一体”城镇体系推进新型城镇化，形成城乡一体、组群发展模式。落实鼓励政策，创新资金投入机制，综合运用转移支付、以奖代补、集中专项等政府性资金，引导社会资金投入特色镇和新社区建设。扎实推进城乡基础设施“六网”工程建设，大力推进已规划的106个新社区建设，因地制宜地推动“强点、连线、扩面”。推进“清洁乡村”工程，加快建设城乡一体垃圾、污水处理体系。同步推进新社区、新产业协调发展，推动城市服务向农村延伸，通过产业带动、城乡服务对接，引导农民转变生产生活方式，促进农民就地转移和农民市民化。二是大力推进农产品加工转化。择优扶持龙头加工企业，带动农产品精深加工、转化增值，提升农产品加工产值占农业产值的比重。支持华容省级农产品加工园与鄂州开发区基础设施对接，引导项目向园区集中，培育引进龙头企业，力争园区过亿元的龙头企业达到5家，新增农产品加工产值10亿元。大力发展特色品牌，做响做大“武昌鱼”等水产品牌，把品牌优势转化成市场优势。三是进一步增强农村发展活力。充分发挥市农业发展投资公司投融资平台和农村综合产权交易平台作用，推动农村资产资本化。用工业理念谋划农业，进一步围绕水产、畜禽、蔬菜、林果四大特色板块培龙头、扩规模、上档次、提效益。强力推进农村土地流转，加快发展龙头企业、种养大户、家庭农场，提高土地规模经营水平，重点打造百里长港、樊寺线和环梁子湖三条都市农业示范带。加强农业基础设施建设，抓好中省投资4.03亿元的“十大重点水利工程”，推进土地整理、农电完善、高效节水排灌、中小河流整治、水库除险加固、池田林路等设施建设。强化农业生产科技服务，推进农业科技化、标准化、机械化生产。加快推进开发式扶贫，加强农民负担监管，落实好各类支农补贴和扶持政策。加快农产品现代流通体系和农村消费服务体系建设和监管。支持发展特色“农家乐”、家庭农场和专业合作社，拓展农民增收渠道。

　　（四）深入推进综合改革，不断增强发展动力。按照三年行动计划总体框架，巩固扩大综合改革成果，推进“五化联动”。一是突出行政管理高效化。巩固葛店行政层级改革试点成果，整合功能区所在地行政职能与公共资源，适时启动功能区改革；推动行政审批制度改革和电子监察制度向区以下扩展；推进事业单位分类改革；推动行政事业单位后勤管办分离、政企分开、统一管理、社会化改革，加快构建精简高效的行政管理体系。二是突出要素配置市场化。全面深化农村产权制度改革，规范建设用地指标生成和交易流转，加快推进“五权”抵押融资，以产权流转带动劳动力、资本、技术、信息等生产要素在城乡之间流动，为建设农村新社区、发展产业化提供外部支撑。以消化盘活低效和闲置土地资产为重点，进一步构建节约集约用地新机制。开展行政事业性国有资产管理体制改革，推动城市公共资源资产化、资产资本化，壮大政府投融资平台，提高城市建设融资能力。加强公共资源交易市场建设，实现交易市场化。三是突出公共资源均衡化。推进“数字鄂州”、“智慧城市”建设，建立覆盖全市的公共地理信息平台；明确农村各类基础设施产权主体，加强监管维护，建立完善的农村基础设施管理运营长效机制；建立健全均衡城乡的公共服务财政政策，加快“五个全民”步伐；创新公共服务管理与运行机制，推动公共服务集中规范管理，引导农民向新城和新社区集中。四是突出社会管理一体化。以标准化和信息化为主要抓手，全面开展农村示范社区创建活动，加快推进电子政务进社区、网格化管理，创新基层组织建设，建立健全以基层民主自治、公共服务和综治维稳融合互动为主要特征的农村基层治理新机制。开展好全国第三次经济普查工作，摸清经济发展“家底”。五是突出发展模式“两型”化。坚持硬件建设和制度创新相结合、政策支持和市场运作相结合，建设一批在全省有影响的“两型”示范农村社区和示范路；建立健全产业“两型”化财政、土地激励政策；建立健全城乡生态环境综合整治财政政策，全力争取上级建立梁子湖生态补偿机制，探索转移支付、专项补贴、异地开发等多样化的生态补偿方式；建立与科学发展观相适应的领导干部综合实绩考核体系，着力推进生产方式、生活方式“两型”化。

　　（五）不断完善城市功能，提升宜居宜业水平。以建市30周年为契机，加快实施一批重大工程，展现鄂州崭新的城市面貌。一是加强全域规划管理工作。继续深化、细化、优化全域规划编制体系，加快编制城乡总体规划以下各类规划，编制完成与之衔接的产业发展、基础设施、社会事业、生态环境等各行业专项规划。坚持“一张图规划、一支笔审批、一盘棋建设、一张网管理”，理顺市区规划管理体制，理顺规划监督机制，强化城乡规划的引导、协调、统筹、管控作用，形成有序布局、集约优化、科学发展的良性局面。二是推进重大功能设施建设。继续发行城投债，多渠道筹措建设资金。筹资50亿元推进主城区和各区、开发区基础设施建设。加快吴楚大道、鄂州大道、鄂黄高速等城市交通骨干路网建设，启动重载车专用通道建设，力争鄂咸高速今年开工，加快三江港鄂州铁路北站工程建设，建成主城客运综合枢纽站和站前广场，确保城际铁路今年底通车。启动葛华新城客运站、梁子湖区旅游客运站建设。加快江碧路等主次街道升级改造。建成寒溪生态广场、鄂州大道绿化景观工程。启动城市展示馆、城南吴都中央公园、葛山文化体育园工程。建成葛华新城20万吨水厂。三是提升城市管理水平。构建与城乡网格化管理相衔接的综合管理体系，继续开展“四城创建”活动，改进窗口行业服务质量，加强市政道路、绿化、环卫、照明、供排水、强弱电等设施管护，加强对重点区域、薄弱地段、苗头性问题进行专项治理，形成常态机制。严控违法建设，整治脏乱差，确保市容整洁有序。四是推进生态文明建设。加快建设“灵秀鄂州”。加大工业污染的防治，严控工业污染源。突出湖泊生态、林地、湿地、山体、水体保护，实行最严格的保护措施。抓紧实施洋澜湖综合整治和生态修复。力争梁子湖第二入江通道核准，统筹谋划梁子湖、花马湖、鸭儿湖、南迹湖四大水系治理与生态恢复。加快推进新能源示范城市建设。（六）坚持保障和改善民生，确保社会和谐稳定。一是大力发展社会事业。推进义务教育均衡发展，华容区、梁子湖区要通过省评估验收。发展各类教育，建设城南幼儿园，启动中小学校外综合实践基地建设。开展鄂州职业大学政校行企联建。深化公立医院改革，加快市中心医院、儿童医院、梁子湖区医院和各区急救中心等医疗机构建设，按照标准化要求完善乡镇卫生院、村卫生室和社区卫生服务机构设施。加强文化基础设施建设，继续推进文化惠民和全民健身工程向广度延伸。实现全市村级农家数字书屋全覆盖。综合治理出生人口性别比。二是完善社会保障体系。坚持就业优先战略，不断推进全民创业，争创全国创业型城市。完善社会保险体系，推进城乡一体的养老、医疗保险体系建设，稳步扩大参保率。推进事业单位养老保险制度改革。提高社会保障信息化管理水平，实现社会保障“一卡通”。加强社会救助，继续提高城乡低保标准，建设市民政服务中心。加强保障性住房建设和管理，缓解住房困难群众基本需求。加强非公企业住房公积金制度建设。三是加强社会安全防控。加强社会治安防控体系建设，提高人民安全感。完善安全生产责任制，推进消防安全管理创新，加强隐患排查整改，提高安全防范和全民消防意识。完善体制，加强生产和流通环节监管，保障食品药品等消费安全。加强信访工作，畅通社情民意诉求渠道。加强矛盾排查、化解和应急处置工作，维护和谐稳定。四是扎实办好“十件实事”。逐项落实责任部门和责任人，制订科学的工作方案，倒排工作进度，向社会公开，接受社会监督。确保实事办实，好事办好。（七）加强政府自身建设，打造人民满意政府。认真学习贯彻党的十八大精神，提升精神区位，搞好政府自身建设。一是切实转变作风。大兴调查研究之风，认真落实有关转变作风的各项规定，加强监督检查。切实改进会风、文风、话风、事风。落实首问负责、限时办结等制度，在市直政府部门推行AB角工作制度，并向社会公开。加强政务服务体系建设，提升政务服务水平。推进机关事务统一集中管理，深化机关服务社会化改革，厉行勤俭节约，大力压减“三公”经费。二是推进法治政府建设。提高领导干部运用法治思维和法治方式深化改革、推动发展、化解矛盾、维护稳定能力。完成市直部门行政职权清理，限制自由裁量权，确定职权边界。加强能力席位建设。深入开展相对集中行政复议权试点。改进行政执法，着力解决少数部门履职越位、错位、缺位等问题。启动政府绩效管理监察，提高行政效率。自觉接受人大及其常委会的工作监督、法律监督和政协的民主监督，办好建议提案。认真听取民主党派、工商联、无党派人士意见，支持工青妇等人民团体更好地发挥作用。三是坚持廉洁从政。认真落实党风廉政责任制，切实履行“一岗双责”。抓好源头治腐，开展制度廉洁性评估工作。大力推行政务、村务、厂务和事务公开，让权力在阳光下运行。加强对工程建设、政府采购、招投标、资金运用等重点领域和重要环节的监管。强化廉政监察和审计监督，注重审计结果的运用。加强政风行风建设，坚决纠正损害群众和企业利益的不正之风，坚决查处损害经济发展环境的行为，坚决治理“庸、懒、散、奢”，做到为民、务实、清廉，树立廉洁高效的政府形象，努力建设人民满意的政府。各位代表，鄂州的发展已经进入一个新的关键时期，新的一年任务艰巨而繁重。让我们在十八大精神的指引下，在省委、省政府和市委的坚强领导下，坚定信心，团结奋进，开拓创新，为全面推进“五个鄂州”和宜居宜业组群式大城市建设，率先在全省建成小康社会而努力奋斗！
